# Effects of *Dangguixu-san* on acute lateral ankle sprain: study protocol for a randomized controlled trial

**DOI:** 10.1186/s13063-018-2571-1

**Published:** 2018-03-27

**Authors:** Jae-Hong Kim, Eun-Yong Lee, Myung-Rae Cho, Cham-Kyul Lee, Ji-Hyun Cho

**Affiliations:** 1 Department of Acupuncture and Moxibustion Medicine, College of Korean Medicine, Dong-Shin University, Naju City, 58245 Republic of Korea; 2 Department of Acupuncture and Moxibustion Medicine, Semyung University Korean Medicine Hospital in Chungju, Chung-Ju, 27427 Republic of Korea; 3 Department of Social Welfare, College of Health and Welfare, Dong-Shin University, Naju City, 58245 Republic of Korea; 4 Department of Acupuncture and Moxibustion Medicine, Dong-Shin University Gwangju Oriental Hospital, 141, Wolsan-ro, Nam-gu, Gwangju City, 61619 Republic of Korea

**Keywords:** Ankle sprain, *Dangguixu-san*, Randomized controlled trial, Study protocol, Double-blind

## Abstract

**Background:**

Ankle sprain is a common musculoskeletal injury. In Korean medicine, blood stasis is thought to be the main cause of pain and swelling in patients with ankle sprain. *Dangguixu-san* (DS), a herbal extract, is widely used in Korean medicine for the treatment of traumatic ecchymosis and pain by promoting blood circulation and relieving blood stasis. However, the effects of DS on ankle sprain have not been evaluated in a randomized clinical trial. Here, we describe the protocol for a randomized controlled trial that will evaluate the efficacy and safety of DS for the treatment of ankle sprain.

**Methods/design:**

In this randomized, double-blinded, placebo-controlled, parallel-arm clinical trial with a 1:1 allocation ratio, participants (*n* = 48) with acute lateral ankle sprain (ALAS) that occurred within 72 h before enrollment will be randomly assigned to a DS (*n* = 24) or a placebo (*n* = 24) group. Both groups will receive acupuncture treatment once a day for 5 days a week (excluding Saturday and Sunday) and the trial medication (DS/placebo capsule) three times a day for seven consecutive days. The primary outcome measure will be pain relief evaluated using a Visual Analog Scale (VAS). Secondary outcome measures will include Foot and Ankle Outcome Scores (FAOS), edema, European Quality of Life Five-Dimension-Five-Level Scale (EQ-5D-5 L) scores, and the number of recurrent ankle sprains. VAS, FAOS, edema, and EQ-5D-5 L scores will be recorded before, at the end of, and at 4 weeks after treatment completion. EQ-5D-5 L scores will be additionally recorded at 26 weeks after treatment completion. The number of recurrent ankle sprains will be recorded at 4, 8, 12, and 26 weeks after treatment completion.

**Discussion:**

This study is expected to provide evidence regarding the efficacy, safety, and usefulness of DS for the treatment of ALAS.

**Trial registration:**

cris.nih.go.kr, registration number: KCT 0002374. Registered on 11 July, 2017 and approved by the Ministry of Food and Drug Safety (registration number, 31244).

**Electronic supplementary material:**

The online version of this article (10.1186/s13063-018-2571-1) contains supplementary material, which is available to authorized users.

## Background

Ankle sprain is one of the more common musculoskeletal injuries and is associated with a high prevalence of persistent problems that lead to an increased burden of chronic health issues in the community [1–5]. The annual incidence rate of ankle sprain ranges from 2.15 per 1000 individuals in the USA (resulting in a cumulative healthcare cost of US$4.5 billion per annum) [1, 2] to 5.3–7.0 per 1000 individuals in Europe [3, 6]. Ankle sprain is commonly considered a benign injury that resolves quickly [7]. However, at least a third of individuals who develop an acute ankle sprain develop chronic ankle instability (CAI) [8–10], which is characterized by residual symptoms, including a feeling of “giving way” and instability, recurrent ankle sprain, and functional loss after acute ankle sprain [11]. CAI not only limits physical activity but also leads to articular degeneration of the ankle joint and an increased risk of osteoarthritis [12]. The three major types of treatment for ankle sprain are surgical treatment, conservative treatment involving immobilization with a plaster cast or splint, and functional conservative treatment with a tape or a semi-rigid or lace-up brace [13].

Blood stasis is an important pathological concept in traditional East Asian medicine (TEAM) since it was first documented in *Huangdi’s Inner Classic*. Generally, blood stasis is a significant pathological product of blood stagnation [14, 15]. Blood stasis occurring within the body is termed blood stasis syndrome (BSS), which is characterized by symptoms such as pain in a fixed position, nyctalgia, dark-purple coloring of the tongue and face, infraorbital darkness, sublingual varicosis, blood spots under the skin or tongue, and an astringent pulse [16]. According to TEAM, many diseases, including ischemic heart disease, cerebrovascular events, diabetes mellitus, chronic gastritis, chronic renal failure, chronic hepatitis, trauma, and dysmenorrhea, could be related to BSS [17, 18]. This phenomenon is termed *EoHyeol* in Korean, *Yu Xue* in Chinese, and *Oketsu* in Japanese [19].

In Korean medicine, because it is considered that pain and swelling associated with ankle sprain could be caused by blood stasis, activation of blood circulation is the main treatment principle for ankle sprain [20, 21]. In addition to conventional treatments, complementary and alternative treatment modalities, such as herbal medicine, have been thought to relieve pain, reduce swelling, and help the body restore damaged tissues [22]. Traditional Korean medications usually contain many compounds that act on multiple targets [23]. The combination of multiple drugs is thought to maximize the therapeutic efficacy by facilitating synergistic actions and preventing potential adverse events (AEs). *Dangguixu-san* (DS), which is composed of *Angelicae gigantis radix*, *Paeoniae radix rubra*, *Linderae radix*, *Cyperi rhizoma*, *Sappan lignum*, *Carthami flos*, *Persicae semen*, *Cinnamomum cassia*, and *Glycyrrhizae radix*, promotes blood circulation and relieves blood stasis; therefore, it is the most frequently prescribed herbal formula for the treatment of traumatic ecchymosis and pain [24] and is recommended for the treatment of ankle sprain [25]. This formula is also known as *Dangkwisoo-san* in Korean and *Tokishusan* in Japanese [26, 27].

Acupuncture is one of the more commonly used therapeutic modalities for painful conditions in complementary and alternative medicine, including Korean medicine [28]. A survey reported that 76% of responding American physicians used acupuncture for ankle sprain, and 90% of them considered it to be somewhat effective [29]. In 2009, approximately 2.8 million Korean individuals were diagnosed with an ankle injury, which was the fifth most common reason for visits to Korean medicine clinics. Of these, 1.2 million individuals sought acupuncture treatment [30]. Considering that DS is mainly used with acupuncture in clinical situations, the present study will use acupuncture as the basic treatment.

Although DS is used for the treatment of ankle sprain in Korean medicine, evidence regarding its efficacy is insufficient. Therefore, we designed a randomized, double-blinded, placebo-controlled, parallel-arm clinical trial for investigating the efficacy and safety of DS for the treatment of ankle sprain. The results of this study are expected to provide evidence regarding the usefulness of DS for the treatment of ankle sprain.

## Method/design

### Objective

The objective of this study is to compare the efficacy of DS treatment combined with acupuncture with that of placebo combined with acupuncture for pain reduction in patients with acute lateral ankle sprain (ALAS) in order to investigate the efficacy of DS for the treatment of ankle sprain.

### Hypothesis

Our null hypothesis is that the pain control effects of DS are not superior to those of placebo in patients with ALAS.

### Study design

The present study design is in accordance with the Standard Protocol Items: Recommendations for Interventional Trials (SPIRIT) and Consolidated Standards Of Reporting Trials (CONSORT) 2010 guidelines [31, 32] (see Additional file 1). The study is a randomized, double-blinded, placebo-controlled, parallel-arm, single-center (Semyung University Korean Medicine Hospital in Chungju, Republic of Korea) clinical trial with a 1:1 allocation ratio. A total of 48 participants who meet the inclusion and exclusion criteria will be randomly allocated to a DS (*n* = 24) or a placebo (*n* = 24) group. Patients in both groups will receive acupuncture treatment once a day for 5 days a week (excluding Saturday and Sunday) and the trial medication (DS/placebo capsule) three times a day for seven consecutive days. The primary outcome measure will be pain relief evaluated using a Visual Analog Scale (VAS). The secondary outcome measures will be Foot and Ankle Outcome Scores (FAOS), edema, European Quality of Life Five-Dimension-Five-Level Scale (EQ-5D-5 L) scores, and the number of recurrent ankle sprains. VAS, FAOS, edema, and EQ-5D-5 L scores will be assessed before, at the end of, and at 4 weeks after treatment completion. EQ-5D-5 L scores will be additionally recorded at 26 weeks after treatment completion. The number of recurrent ankle sprains will be recorded at 4, 8, 12, and 26 weeks after treatment completion.

**Fig. 1:**
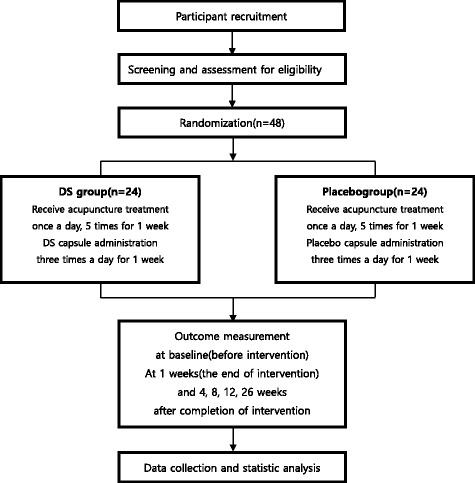
Study design flow chart

This study protocol complies with the principles of the Declaration of Helsinki and Korean Good Clinical Practice guidelines and has been approved by the Ministry of Food and Drug Safety (registration number, 20160318110). The trial has been registered at cris.nih.go.kr (registration number, KCT 0002374). The study design is summarized in Table 1 and Fig. 1.

**Table 1:** Treatment schedule and outcome measures

	Study period										
	Enrollment	Allocation	Post allocation								Close-out
Timepoint	Screening		Visit1	Visit2	Visit3	Visit4	Visit5	Visit6	Visit7	Visit8	Visit9
	Week		1					5	8	13	27
Enrollment											
Informed consent	X										
Sociodemographic profile	X										
Medical history	X										
Vital signs	X	X	X	X	X	X	X	X			
Inclusion/exclusion criteria	X										
Allocation		X									
Clinical laboratory tests	X						X				
Interventions											
Acupuncture treatment			X	X	X	X	X				
Trial medication prescription			X								
Assessments											
Change of medical history			X	X	X	X	X	X	X	X	X
Safety assessment			X	X	X	X	X	X			
Visual Analog Scale of pain			X				X	X			
Foot and Ankle Outcome Score			X				X	X			
Edema of ankle sprain			X				X	X			
European Quality of Life 5-Dimension-5-Level Scale			X				X	X			X
Number of recurrent ankle injuries								X	X	X	X

### Participant recruitment

Participants will be recruited at Semyung University Korean Medicine Hospital in Chungju, Republic of Korea. The study will be advertised through local newspapers, the Internet, and posters in communities and hospitals. Participants will be provided with an explanation about the study by the clinical research coordinator (CRC) and will be requested to voluntarily sign an informed consent before participation. The CRC will continuously monitor the medical condition of the enrolled participants to ensure adherence to the intervention protocols.

### Inclusion criteria

Patients aged older than 19 years who have sustained a grade I or grade II ALAS within 72 h before enrollment and are willing to voluntarily sign an informed consent form will be considered for enrollment. A grade I ankle sprain will be diagnosed when there is no loss of function or ligament laxity (*i.e.*, negative anterior drawer and talar tilt test results), little or no hemorrhaging, no point tenderness, decreased total ankle motion by ≤ 5°, and a swelling of ≤ 0.5 cm. Patients with some loss of function, positive anterior drawer test findings (indicating anterior talofibular ligament involvement), negative talar tilt test findings (indicating no calcaneofibular ligament involvement), hemorrhaging, point tenderness, decreased total ankle motion by > 5° but < 10°, and a swelling of > 0.5 cm but < 2.0 cm will be diagnosed with grade II ALAS [33].

### Exclusion criteria

Subjects with a poor general condition and those who are not fit for acupuncture or DS treatment will be excluded. Other exclusion criteria are as follows: fracture confirmed on x-rays or grade III ankle sprain (which will be defined as the near total loss of function, positive anterior drawer and talar tilt test findings, hemorrhaging, extreme point tenderness, decreased total ankle motion by > 10°, and a swelling of > 2.0 cm) [33]; a history of fracture in the same ankle during the previous year; serious disease conditions (*e.g.*, cancer, kidney, liver, and central nervous system diseases, dementia, and blood clotting abnormalities such as hemophilia); motor or sensory disturbance due to nervous system disorders in the same leg; pregnancy or breastfeeding; medication with drugs such as nonsteroidal anti-inflammatory drugs (NSAIDs), pain relievers, or steroids for pain relief from the time of trauma to participation in the clinical trial (excluding the use of adherent inflammatory pain relievers on the day of screening); impaired hepatic (alanine aminotransferase level, ≥ 80 IU/L) or renal (creatinine level, ≥ 2 mg/dL) function; ineligibility for participation in the trial because of a history of gastrointestinal diseases that might affect the absorption of the trial medication, as judged by the principal investigator (PI); genetic conditions such as galactose intolerance, Lapp lactase deficiency, and glucose-galactose malabsorption; a history of hypersensitivity to the components of the trial medication; and participation in other clinical trials within 4 weeks of screening for the present study or concurrent participation in other clinical trials.

### Ethical considerations

This study has been approved by the Institutional Review Board (IRB) of Semyung University Korean Medicine Hospital in Chungju. The purpose and potential risks of this clinical trial will be fully explained to the participants and their families. All participants will be asked to provide written informed consent before participation.

### Randomization and blinding

After the acquisition of written informed consent and completion of baseline measurements, the 48 enrolled participants will be assigned serial numbers generated using a randomization tool (http://www.randomization.com) and randomly allocated to one of the two study groups (*n* = 24 each). The serial number codes will be inserted into opaque envelopes that will be sealed and stored in a double-locked cabinet.

Blinding will be achieved by randomized labeling and prepackaging of DS and placebo capsules, which will be identical in appearance, taste, and smell. The DS and placebo capsules provided by the pharmaceutical company will be labeled with numbers in accordance with the randomization schedule. Investigators will administer these medications in accordance with the randomization number. Randomization and uncovering will be performed by a third-party team of statisticians. Thus, participants, investigators, and outcome assessors will remain blinded to treatment allocation until study completion.

### Implementation

The CRC will generate the allocation sequence, enroll participants, and assign participants to the intervention groups.

### Intervention

Both groups will receive acupuncture treatment once a day for 5 days a week (excluding Saturday and Sunday) and the trial medication (DS/placebo capsule) three times a day for seven consecutive days. Treatment will be administered by Korean medicine physicians with 6 years of formal university training in Korean medicine, a licence to administer treatment, and at least 1 year of clinical experience. To ensure strict adherence to the study protocol, the physicians will receive training together and employ the same techniques.

#### Acupuncture treatment

All participants will receive acupuncture at the ST36, ST41, BL60, BL62, KI3, KI6, GB39, and GB40 points on the affected side [25]. Only sterile, stainless steel, disposable acupuncture needles (size, 0.25 × 30 mm; Dong Bang Acupuncture, Inc., Boryeong, Republic of Korea; product no. A84010.02) with guide tubes will be used. The depth of insertion will be 10–20 mm depending on the location of the needle [34]. After insertion, the needles will be left in position for 15 min in every session. Manual stimulation and electroacupuncture will not be applied (see Table 2).

**Table 2:** Revised STandards for Reporting Intervention in Clinical Trials of Acupuncture (STRICTA)

	Item criteria	Description
1.Acupuncture rationale	1a. Style of acupuncture	Korean Medicine Therapy
	1b. Reasoning for treatment provided – based on historical context, literature sources, and/or consensus methods, with references where appropriate	1. Discussion among four physicians who practice Korean medicine (consensus) 2. Textbook of acupuncture and moxibustion medicine 3. Relevant articles [20] Selection of treatment regions based on textbooks, related papers, and expert discussions
	1c. Extent to which treatment varied	Standardized treatment
2. Details of needling	2a. Number of needle insertions per subject per session (mean and range where relevant)	8
	2b. Names (or location if no standard name) of points used (uni-/bilateral)	ST36, ST41, BL60, BL62, KI3, KI6, GB39, GB40
	2c. Depth of insertion, based on a specified unit of measurement or on a particular tissue level	The depth of insertion is 10 to 20 mm, depending on the location of the needle [21]
	2d. Responses sought	No *de qi* or muscle twitching – only sensation due to needle insertion
	2e. Needle stimulation	None
	2f. Needle retention time	15 min per session
	2 g. Needle type	sterile, stainless, disposable acupuncture needles (size 0.25 × 30mm; Dong Bang Acupuncture, Inc., Boryeong, Republic of Korea; product no: A 84010.02)
3. Treatment regimen	3a. Number of treatment sessions	5
	3b. Frequency and duration of treatment sessions	Five times/week for 1 week, 15 min per session
4. Other treatment components	4a. Details of other interventions administered to the acupuncture group	None
	4b. Setting and context of treatment – including instructions to practitioners – as well as information and explanations given to patients	Practitioner-patient conversation about the context of the treatment, life habits, and daily life management
5. Practitioner background	5a. Description of participating acupuncturists	Korean medicine physician with the following qualifications: 6 years of formal university training in Korean medicine, a license, and at least 1 year of clinical experience
6. Control or comparator interventions	6a. Rationale for the control or comparator in the context of the research question, with sources that justify the choice	Korea Institute of Oriental Medicine. Ankle sprain Korean Medicine Clinical Practice Guideline. Seoul: Elsevier Korea. L.C.C. 2015;163–7
	6b. Precise description of the control or comparator; details for items 1–3 above with the use of sham acupuncture or any other type of acupuncture-like control	Acupuncture combined with a Kinesiotape (AcuKT) group will receive the ankle meridian tendino-musculature and figure-of-eight-shaped form of KT treatment after acupuncture treatment by same practitioner. The KT treatment method will be conducted as follows: first, an I-shaped tape will be applied from ST42 to ST36 over the tibialis anterior muscle. Second, an I-shaped tape will be applied from GB42 to GB34 over the peroneus longus and brevis muscles. Third, an I-shaped tape will be applied from the abductor digiti minimi muscle and wrapped around the ankle in a figure-of-eight shape to the abductor hallucis muscle covering both the medial and lateral malleoli

#### Trial medication

**Fig. 2:**
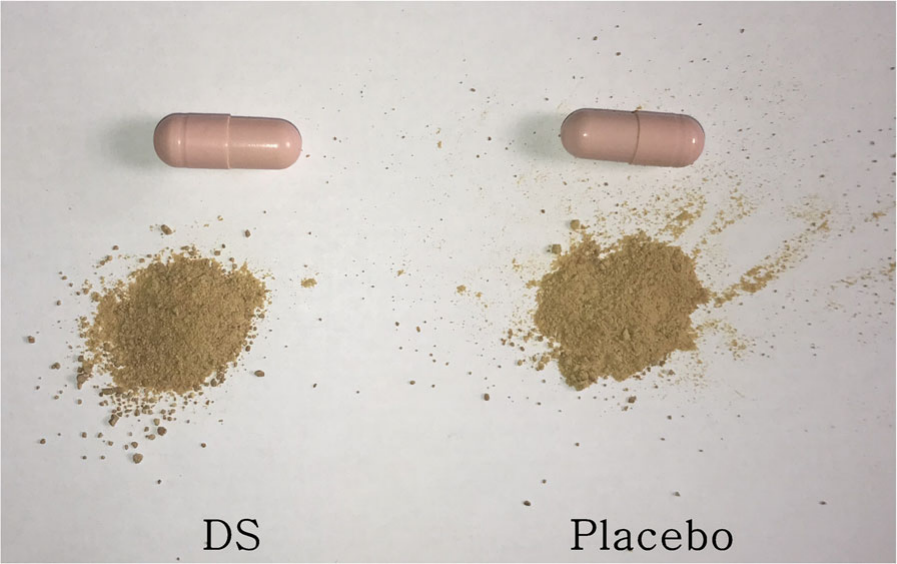
Appearance of *Dangguixu-san* (DS) and placebo capsules used in the study

The trial medications will include DS and placebo capsules, which will be identical in appearance, taste, and smell. The DS formulation composed of granular extracts of nine herbal substances, will be a gray-brown powder contained in a 0.6-g pink capsule (Fig. 2). The placebo will be composed of 300 mg of corn starch, 150 mg of lactose hydrate, 1.5 mg of magnesium stearate, and 48.5 mg of caramel colorant. Both medications will be administered at a dose of three capsules three times daily (total daily dose, nine capsules) for a period of 1 week.

The manufacturing process for the DS capsules is as follows. First, 0.63 g of *Angelicae gigantics radix*, 0.42 g of *Paeoniae radix rubra*, 0.42 g of *Linderae radix*, 0.42 g of *Cyperi rhizoma*, 0.42 g of *Sappan lignum*, 0.33 g of *Carthami flos*, 0.29 g of *Persicae semen*, 0.25 g of *Cinnamomum cassia*, and 0.21 g of *Glycyrrhizae radix* are added to the extractor. The mixture is extracted with 8–10 volumes of purified water at 80–100 °C for 3–4 h. The extract is filtered (100 mesh), concentrated under reduced pressure at ≤ 60 °C, and dried to obtain 350 mg of a granular extract. Then, 45 mg of lactose hydrate, 100 mg of corn starch, and 5 mg of magnesium stearate are mixed into the granular extract, and the mixture is converted into granules, dried, crushed, and filled into capsules.

Capsules will be sourced from Kyungjin Pharmaceutical Co., Ltd. (Icheon, Gyeonggi-do, Republic of Korea (product no. 02190)). The medication will be dispensed as 0.6-g capsules packed in sealed boxes containing a 1-week dose.

Between the preintervention period and 4 weeks after treatment completion, participants will not be allowed to take the following drugs: NSAIDS, pain relievers, steroids, or adherent inflammatory pain relievers; drugs containing potassium, licorice, glycyrrhizic acid, furosemide, ethacrynic acid, or trichloromethazine; and drugs containing any of the main ingredients of DS. Participants will be asked to return their medication boxes to allow enumeration of leftover capsules and for monitoring participant adherence.

### Outcome measurements

VAS, FAOS, edema, and EQ-5D-5 L scores will be assessed before, at the end of, and at 4 weeks after treatment completion. EQ-5D-5 L scores will be additionally recorded at 26 weeks after treatment completion. The number of recurrent ankle sprains will be recorded at 4, 8, 12, and 26 weeks after treatment completion.

#### Primary outcome

Because the objective of this study is to investigate the efficacy of DS therapy for pain relief in patients with ALAS, changes in the pain severity (measured using VAS) will be considered as the primary outcome. The VAS will be a 10-cm straight line marked at each end with the anchor labels “no pain” and “pain as bad as it could be” [35]. Patients will be asked to mark the line at a point representing their severity of pain. Scores will be recorded in millimeters, with a total score range of 0–100 mm [36].

#### Secondary outcomes

Secondary outcomes will include changes in FAOS, edema, EQ-5D-5 L scores, and the number of recurrent ankle sprains. The FAOS is a self-administered questionnaire specific to feet and ankles. It is designed to assess week-to-week changes in symptoms and function after foot and ankle injuries and comprises five subscales: pain (nine items), other symptoms (seven items), activities of daily living (17 items), sports and recreational activities (five items), and foot-and-ankle-related quality of life (four items). The subscales are scored separately using a Likert response format, with higher scores indicating higher levels of function [37].

Edema will be measured in centimeters using the figure-of-eight method. A measurement tape will be applied across the following landmarks in a figure-of-eight fashion: navicular tuberosity, distal tip of the lateral malleolus, distal tip of the medial malleolus, and base of the fifth metatarsal. The resulting value will be compared with the corresponding value for the healthy ankle [38].

The European Quality of Life Five-Dimension Scale (EQ-5D) is a generic instrument for assessment of health-related quality of life. It is based on a descriptive system that defines health in terms of five dimensions: mobility, self-care, usual activities, pain/discomfort, and anxiety/depression. Each dimension has three response categories: no, some, or extreme problems. The EQ-5D-5 L, which will be used in this study, is a new version of the EQ-5D that includes five levels of severity in each of the existing five EQ-5D dimensions [39].

Recurrent ankle sprain will be defined as ankle sprain occurring as a result of participation in sports or other daily activities and causing one or more of the following: discontinuation of the sports activity, inability to fully participate in the next planned sports activity, inability to go to work/school the following day, and the requirement of medical attention (ranging from onsite care administered by a general practitioner to personal care administered by a sports physician) [40].

### Incidence of adverse events

AEs are undesirable and unintentional signs, symptoms, or diseases that appear during or after treatment in a clinical trial. The participants in this study will be required to voluntarily report any AEs. All AEs that occur during the trial will be documented. Adverse events that might occur in this study include skin irritation, urticaria, itching, anorexia, stomach discomfort, diarrhea, nausea, vomiting, pseudoaldosteronism, and hypokalemia. The CRC will record all AEs in detail, including the time and date of occurrence, degree of severity, any measures related to the treatment of the AE, and any potentially causal relationship between the treatment and the AE, and report all AEs to the PI and relevant IRB. In case of serious AEs (SAEs), defined as those causing severe disability or malfunction, appropriate measures will be taken and the incident will be immediately reported to the PI and relevant IRB. In case an AE occurs because of the clinical trial, participants will notify the CRC and PI and will be compensated by the “Clinical Trial Compensation.”

### Quality assurance

This protocol has been reviewed and revised several times by experts on acupuncture, herbal medicine, orthopedics, statistics, and methodology. Before the trial, all researchers will be requested to attend a series of training sessions, which will ensure that the personnel involved fully understand the trial protocol and standard operating procedures (SOPs) that will be employed during the study. The Data Monitoring Committee will be composed of the PI and CRC. The clinical trial will be monitored by a clinical research associate (CRA), who will check all documents related to the clinical trial, including Case Report Forms (CRFs) and SOPs, and ensure that the clinical trial is conducted in accordance with the prescribed protocols and SOPs. Monitoring will be conducted independent of the PI. In the event that the protocol described herein is revised, the revisions will require approval from the Ministry of Food and Drug Safety and the IRB of Semyung University Korean Medicine Hospital in Chungju.

### Sample size estimation

Because of the lack of adequate preliminary studies, we adopted a pilot study design with 24 participants in each group, considering the limited research funds, study period, and recruitment opportunities.

### Statistical analysis

Baseline characteristics will be compared between the two groups. Repeated measures analysis of covariance (RM ANCOVA) will be performed to determine differences between groups, considering the different baseline characteristics (covariance). Continuous data will be presented as means and standard deviations and compared using the independent *t* test or Wilcoxon’s rank sum test, while categorical data will be presented as frequencies and percentages and compared using the chi-square or Fisher’s exact test. VAS scores for pain and the secondary outcomes (FAOS, edema, and EQ-5D-5 L scores) will be evaluated by RM ANCOVA. Dependent variables will include values measured before, at the end of, and at 4 weeks after treatment completion. Changes in the VAS score from baseline to treatment completion (visit 5) and 4 weeks after treatment completion (visit 6) will be determined by the paired *t* test. A repeated contrast test will be performed to account for time differences in each group. A *P* value of < 0.05 will be considered statistically significant. We will perform per-protocol analysis for the assessment of efficacy and a supplementary full analysis set. All statistical analyses will be performed using SPSS version 22.0 (SPSS Inc., Chicago, IL, USA).

Data for subjects who meet the dropout criteria (*i.e.*, < 80% compliance with the protocol, incidence of SAEs, reluctance to continue the trial, incomplete data that could influence the trial, large error in protocol or significant deviation in implementation, and decision to terminate trial participation by the PI or IRB) will be excluded. Missing values will be implemented by multiple imputations. In addition, differences between subjects who complete the study and dropouts will be statistically analyzed to determine whether any particular factor is significantly associated with participation dropout. Interim analyses will not be performed.

### Confidentiality and data management

Identification records of the participants will be kept confidential until publication of the results of the study. All documents related to the trial, including CRFs, will be recorded and labeled with participant identification codes and will not show the name of the participant. These serial number codes will be stored in sealed, opaque envelopes and kept in a double-locked cabinet. All participant data will be recorded in Excel files by the CRC. In additionally, raw data (CRFs) will be stored in a cabinet until the end of the study. Written informed consent for the publication of individual details and accompanying images will be obtained from the participants.

## Discussion

The design of this study, including the treatment and evaluation schedules, is based on the designs of several acupuncture studies for ankle sprain [30].

There is good evidence to support the usefulness of immobilization and, occasionally, surgical correction for the management of grade III ankle sprain. However, clinical standards for the acute management of grade I and grade II ankle sprain are not well defined [4]. Therefore, we will include subjects with grade I and grade II ankle sprain in the present study and exclude those with grade III ankle sprain. We will also exclude subjects who are using analgesics that could affect the outcomes of this study and those at risk of AEs associated with DS treatment.

We will use VAS scores for pain as the primary outcome measure and FAOS, edema, EQ-5D-5 L scores, and the number of recurrences as secondary outcomes in order to evaluate the efficacy of DS for relieving pain and swelling, aiding the recovery of ankle function, improving quality of life, and decreasing the number of recurrences in patients with ALAS.

This study has some limitations. First, there is concern that the short treatment period planned for minimizing the dropout rate could affect the results of this study. Second, because preliminary data for determining the sample size is inadequate, we designed this trial as a single-center pilot study. Third, in previous studies [41–44], recurrences were followed up for 52 weeks; however, because of the short study duration, we have scheduled a follow-up period of only 26 weeks.

Nevertheless, the results of this study are expected to provide preliminary evidence regarding the usefulness and acceptability of DS treatment for the treatment of grade I and grade II ankle sprain and serve as a basis for further research.

### Dissemination policy

We will report the final data to the Ministry of Health and Welfare through the Korea Health Industry Development Institute. We will also publish the results after study completion.

### Trial status

This trial is ongoing. Enrollment and trial procedures are expected to be complete by the end of May 2019.

## Additional file

Additional file 1: SPIRIT Checklist. (DOCX 367 kb)

## Abbreviations

AE: Adverse event
ALAS: Acute lateral ankle sprain
BSS: Blood stasis syndrome
CAI: Chronic ankle instability
CONSORT: Consolidated Standards Of Reporting Trials
CRA: Clinical research associate
CRC: Clinical research coordinator
CRF: Case Report Form
DS: *Dangguixu-san*
EQ-5D-5 L: European Quality of Life Five-Dimension-Five-Level scale
FAOS: Foot and ankle outcome score
IRB: Institutional Review Board
NSAID: Nonsteroidal anti-inflammatory drug
PI: Principal investigator
SAE: Serious adverse event
SOP: Standard operating procedure
SPIRIT: Standard Protocol Items: Recommendations for Interventional Trials
TEAM: Traditional East Asian medicine
VAS: Visual Analog Scale
